# Dr. Charlotte E. Mastin

**Published:** 1905-07

**Authors:** 


### OBITUARY.

Dr. Charlotte E. Mastin, a graduate of the University of Buffalo, 1897, died at Wellsboro, Pa., May 20, 1905, aged 33 years.

